# Prevention and Management of Type 2 Diabetes and Metabolic Syndrome in the Time of COVID-19: Should We Add a Cup of Coffee?

**DOI:** 10.3389/fnut.2020.581680

**Published:** 2020-10-06

**Authors:** Sabina Semiz, Fadila Serdarevic

**Affiliations:** ^1^College of Medicine and Health Sciences, Khalifa University, Abu Dhabi, United Arab Emirates; ^2^Association South East European Network for Medical Research-SOVE, Sarajevo, Bosnia and Herzegovina; ^3^Department of Child and Adolescent Psychiatry, Erasmus Medical Centre Rotterdam, Rotterdam, Netherlands

**Keywords:** polyphenols, coffee, COVID-19, ACE2, type 2 diabetes, obesity, insulin resistance, physical activity

## Abstract

Recent evidence shows that COVID-19 patients with existing metabolic disorders, such as diabetes and metabolic syndrome, are exposed to a high risk of morbidity and mortality. At the same time, in order to manage the pandemic, the health authorities around the world are advising people to stay at home. This results in decreased physical activity and an increased consumption of an unhealthy diet, which often leads to an increase in body weight, risk for diabetes, insulin resistance, and metabolic syndrome, and thus, paradoxically, to a high risk of morbidity and mortality due to COVID-19 complications. Here we summarize the evidence demonstrating that the promotion of a healthy life style, including physical activity and a dietary intake of natural polyphenols present in coffee and tea, has the potential to improve the prevention and management of insulin resistance and diabetes in the time of COVID-19 pandemic. Particularly, it would be pertinent to evaluate further the potential positive effects of coffee beverages, rich in natural polyphenols, as an adjuvant therapy for COVID-19, which appear not to be studied sufficiently.

## Introduction

As we have just approached the 10 millionth case of the novel coronavirus disease (COVID-19) worldwide, an increasing amount of evidence has been accumulating to indicate its poorer prognosis in patients with diabetes and hypertension, who appear to be exposed to the greater risk of the severe morbidity and mortality (1–3). A recent meta-analysis demonstrated that the incidences of diabetes and hypertension were about two-fold higher, and cardio- and cerebrovascular diseases were three-fold higher in severe/intensive care unit (ICU) cases as compared to non-ICU severe cases (4). Similarly, another recent report showed that the triglyceride and glucose index (TyG) was associated with an increased risk of morbidity and mortality in COVID-19 patients, suggesting that this marker of insulin resistance could also be a valuable marker for identifying a poor outcome of coronavirus infection (5).

Human pathogenic coronaviruses, including severe acute respiratory syndrome coronavirus [SARSCoV] and SARSCoV-2, enter cells upon binding through angiotensin-converting enzyme 2 (ACE2), a recently characterized monocarboxypeptidase and the first ACE homolog. The ACE-2 receptor is a part of the renin-angiotensin-system (RAS), consisting of classical ACE-Ang-II-AT1R axis and non-classical, recently discovered ACE-2-(A1-7)-Mas axis. The enzyme ACE2 generates additional RAS peptides, such as Angiotensin-1-7 (A1-7), which is associated with the unique functions of this multifaceted system (6). Interestingly, an upregulation of ACE-Ang-II-AT1R axis leading to pro-inflammatory effects, insulin secretory defects and increased insulin resistance was demonstrated in people with underlying lipid and other metabolic disturbances, such as in the case of metabolic syndrome and diabetes (2). In parallel, there is a downregulation of ACE-2-(A1-7)-Mas axis and thus, diminished anti-inflammatory effects and protection against pancreatitis and insulin resistance. It is proposed that the already distraught ACE-2-(A1-7)-Mas in diabetes/insulin resistance is additionally strained due to the virus's use of the ACE-2 to enter the host cell (2).

ACE2 is expressed in the lung, intestine, kidney, blood vessels, cardiomyocytes, immune and other cells (7–12). Since ACE2 is expressed in the liver (13), skeletal muscle (14), and adipose tissue (15), it is proposed that by being present in these insulin sensitive organs, the ACE2 may have a role in regulating insulin sensitivity and glucose homeostasis (16). ACE2 expression is reduced in patients with Type 2 Diabetes (T2D) and kidney dysfunction (17, 18). It was also suggested that decreased expression of ACE2 and use of RAS system antagonists in diabetes management may contribute to poor prognosis in COVID-19 patients (19). Furthermore, *ACE2* genetic variants are reported to be associated with T2D, hypertension, dyslipidemia, carotid arteriosclerosis and left ventricular remodeling (20). It was suggested that *ACE2* polymorphisms may be used as novel risk markers for the left ventricular hypertrophy (LVH) in hypertensive patients (21) and *ACE2* variation was indicated to be associated with risk for cardiomyopathies (22). Indeed, recent COVID-19 cases demonstrated cardiac dysfunction as a serious complication and prognostic tool (23).

Previous studies have shown that ACE2 knockout mice had impaired glucose tolerance or diabetes, as well as dysfunction of endoplasmic reticulum and mitochondria in skeletal muscle that could be improved by ACE2 activation (24). Interestingly, Takeda et al. (16) demonstrated a beneficial role of ACE2 in T2D management and a protective role against caloric overload, likely via regulation of GLUT4 expression and glucose uptake (16). It was also shown that the activation of the ACE2/(A1-7)/Mas axis can improve hepatic insulin resistance, increase glucose uptake and decrease glycogen synthesis in the liver (25), therefore ACE2 was suggested to be a novel drug target for treating insulin resistance (26).

ACE2 enzyme has been also identified in pancreatic islets and it was recommended to monitor serious COVID-19 cases for the pancreas damage (27). A protective role of ACE2 in the pancreas was suggested based on the recent findings of an increased ratio of dedifferentiated beta cells in ACE2-knockout mice under high-fat diet and consequent insulin resistance, which was improved after the administration of A1-7 (28). A recent review outlined several protective effects of the A1-7 mimetics observed in animal studies, including protection from atherogenesis, cardiovascular dysfunction, and cardiac hypertrophy (29). It was shown that high glucose levels can activate the ACE2/(A1-7)/Mas and APN/Ang IV/IRAP RAS axes simultaneously with increased insulin secretion in the rat insulinoma cell line (30). Frantz et al. (31) demonstrated that enalapril treatment protected against the body mass increase, normalized the islet morphology, function, and beta cell mass, thus preserving the pancreatic beta-cell function probably by activating the ACE2/(A1-7)/Mas receptor axis.

## Physical Activity and COVID-19

Due to the COVID-19 pandemic, a large number of people around the world have been in self-isolation or quarantine for several weeks or months. In addition, social activities have been discouraged, unhealthy diets are being consumed in higher amounts, while exercise facilities, national parks, and playgrounds have been closed. A recent study examined adults' physical activity during the COVID-19 imposed confinement in Belgium, and found that most of the people participating in the study reported to exercise less and sit more during the lockdown (32). Another study performed in the USA showed that 48% of obese patients exercised less, 50% increased food stockpiling and about 60% increased stress-related eating, while 73% of them reported increased anxiety and 84% augmented depression since the confinement was initiated (33). A recent USA survey indicated that about 90% of responders spent more time at home and 22% gained 5–10 pounds, mostly due to absence of dietary restraint, stress-stimulated eating, and reduced physical activity imposed by COVID-19 confinement (34). Furthermore, an Italian survey performed in April 2020 indicated that weight gain was observed in almost 50% of the population, despite some positive self-implemented life style modifications (35). A recent international online survey, performed in Asian, African and European populations, reported the negative effect of COVID-19 home confinement on all physical activity intensity levels, including vigorous, moderate, and walking activities, as well as showed that daily sitting time increased from 5 to 8 h per day, along with unhealthier food consumption and meal plan (36).

Recent studies explored the health impacts of a prolonged reduction in physical activity and overeating under circumstances of home confinement and found that increased total body fat and elevated levels of inflammatory cytokines appear to be major factors in exacerbation of insulin resistance (1). Another unhealthy consequence of COVID-19 imposed home confinement is the limited exposure to daily light, which can lead to decreased levels of Vitamin D, essential to perturb viral cellular infection by interacting with ACE2 cell entry receptors (37). In addition to the well-established association between vitamin D deficiency and insulin resistance and T2D risk (38), recent publications suggest its association with COVID-19 severity (39, 40).

## Management of Insulin Resistance in the Time of COVID-19

In order to combat the undesirable changes in the lifestyle and dietary habits adopted during the current COVID-19 pandemic, personalized lifestyle modifications, including home exercising and consuming healthy food, are being promoted. A recent study recommended regular exercise, together with a modest (20–25%) reduction in caloric intake, for protecting cardiovascular function (41) and inducing positive immunomodulatory effects (42). Furthermore, exercise-induced immunomodulation might be a key tool in improving immune responses against the SARS-CoV-2 infection. This is in line with the well-established intervention to increase physical activity and reduce body weight as the first-line recommendation in preventing and treating insulin resistance, (pre)diabetes, metabolic syndrome, and other related disorders (43), which apparently has a high potential to diminish risk of severe form of COVID-19.

In addition to stimulating healthy lifestyle choices, the management of insulin resistance, pre-diabetes and T2D is supported by pharmacological treatment, with multiple targets for preventive and treatment interventions. As described earlier, the key components in SARSCoV-2 viral entry are ACE2 receptor for cell entrance and the serine protease TMPRSS2 for the proteolytic cleavage of viral spike (S) protein (44). Recently, it was shown that T2D patients had increased plasma total protease and serine protease activities (45). Interestingly, plasma serine protease activity was positively associated with levels of hemoglobin A1c (HbA1c), body mass index (BMI) and homeostasis model assessment-insulin resistance (HOMA-IR) index, thus, supporting the potential role of serine protease in development of T2D and offering novel drug targets for T2D management (45). Furthermore, it was proposed that the already available recombinant ACE2 may be employed for treating COVID-19 (46).

Evidence has been accumulating to demonstrate the beneficial effects of metformin, the first-choice oral anti-diabetic drug, in treating several other aging-related diseases, including obesity, pre-diabetes, metabolic syndrome, cardiovascular disease, non-alcoholic fatty liver disease (NAFLD), cancer, cognitive decline (47), as well as immunomodulatory, and other defects (48–50). Interestingly, a recent study performed in the Tongji Hospital of Wuhan in China showed that diabetes treatment with metformin was associated with decreased mortality as compared to diabetics not treated with this medication (51). Furthermore, it was suggested that metformin may have an inhibitory effect on the virus, through increased insulin sensitivity (52). Another study performed in the patients older than 65 years of age, who were hospitalized with pneumonia and with a history of diabetes, found that the preceding metformin treatment was associated with a markedly lower death rate in those patients (53). In line with this, it was recently suggested to use metformin as an adjuvant therapy in older, obese, and diabetic patients with COVID-19, who would benefit from this treatment through the reduction of weight, pneumonia, and protrombotic events, partially through the metformin's ability to prevent inflammation and decrease high circulating levels of cytokines, such as interleukin-6 (IL-6) (54). However, another recent paper cautioned that in patients with severe forms of COVID-19, who are exposed to the risks of lactic acidosis and ketoacidosis, metformin and sodium-glucose co-transporter-2 (SGLT2) inhibitors should be discontinued (55).

Furthermore, a recent study showed that pioglitazone treatment significantly increased the serum levels of ACE2, angiotensin-(1-7) and the hepatic ACE2 expression (56). Pioglitazone belongs to the class of oral antidiabetic drugs, thiazolidinediones (TZD), which were introduced about 10 years ago and widely used to treat insulin resistance and T2D until recently, when discontinued due to increased cardiovascular risk associated with rosiglitazone administration (57). However, TZDs use for the treatment of other diseases has been revaluated in regards to their anti-inflammatory properties (58), including their potential use in treating COVID-19.

## Beneficial Effects of Dietary Polyphenols in COVID-19 Management

A growing evidence from epidemiological studies demonstrated that dietary polyphenols might be used to treat and prevent T2D obesity, and insulin resistance (59). Polyphenols from the grape seed, olive oil, tea, coffee, propolis, cocoa, and chocolate have been shown to have anti-diabetic effects through reducing insulin resistance, glucose and HbA1c levels (60). Resveratrol is a polyphenol and potent antioxidant found in grape, which demonstrated anti-inflammatory and antiviral activity against several viruses (61). A recent meta-analysis indicated that resveratrol may improve cardiometabolic status and alleviate certain risk factors for cardiovascular disease, including insulin resistance and elevated cholesterol levels (62). Furthermore, previous studies demonstrated that resveratrol can deactivate the renin-angiotensin system (63, 64) and have a protective role by upregulating ACE2 (65). Interestingly, as the recent report suggested, resveratrol can be a promising anti-COVID-19 drug candidate, likely acting through interference with the viral spike protein function (66). This is in line with the results from previous studies, which have shown that resveratrol inhibited the Middle East Respiratory Syndrome coronavirus (MERS-CoV), extended cellular survival after virus infection (67), and demonstrated antiviral activity against other viruses (68).

In addition to resveratrol, other polyphenols also demonstrated a beneficial effect in COVID-19 management. Recent reports summarized diverse natural compounds, with the majority being classified as polyphenols, which demonstrated their potential to inhibit coronavirus in humans (69, 70), as well as other viruses (71–74). The potential effects of curcumin, a natural polyphenolic compound, include inhibition of the entry of virus, its encapsulation, and activity of viral protease, suggesting its therapeutic potential in treating SARS-CoV-2 (75). The major components of tea polyphenols, such as theaflavins, showed anti-HIV effect (76), while catechins demonstrated anti-diabetic (77) and hypocholesterolemic activities (78). Furthermore, tea extracts showed antioxidant activity (79) and beneficial effects on protein oxidation- and glycation-associated diseases (80). Importantly, the tea components also showed antiviral activity against the influenza B, H1N1 and H3N2 influenza viruses, most likely through the inhibiting of virus adsorption and replication (81). It has been shown that green tea extract can be used as natural disinfectant (82, 83). In addition to their virucidal activity against a spectra of viruses, including influenza virus, herpes simplex virus, vaccinia virus, coxsackie virus, poliovirus-1, and human rotavirus, theaflavins also demonstrated the ability to alleviate bovine rotavirus and bovine coronavirus infections (84). The beneficial effects of green tea, black tea, and oolong tea have been well-known for many years and a recent study demonstrated that oolonghomobisflavan-A is a potential bioactive molecule responsible for beneficial tea effects, most likely acting through the inhibition of the main protease of SARS-CoV-2 (85). Three polyphenols, including epicatechingallate, epigallocatechin gallate, and gallocatechin-3-gallate, from green tea were selected as promising drug candidates to treat COVID-19 due to their strong interaction with one or both residues in Cys145–His41 catalytic dyad of the main protease of SARS-COV-2 (86).

## Coffee in the Time of COVID-19

In addition to tea, coffee is another commonly consumed beverage in the world, which appears to increase insulin sensitivity and glucose uptake in skeletal muscle. A recent report identified for the first time that the phytochemicals from coffee and cocoa affect the phosphorylation of the insulin receptor signaling pathway and stimulated GLUT-4 translocation, increasing intracellular glucose utilization (87). In line with this, a systematic review of clinical trials analyzing the effects of coffee consumption on glucose metabolism, indicated an improvement of insulin response and glucose metabolism in long-term studies (88). Previous studies have shown that in animal models chronic caffeine intake prevented and reversed insulin resistance induced by aging and hypercaloric diets (89). Recently, coffee consumption by high-risk diabetic individuals was positively associated with pancreatic beta cell function, so they had lower fasting glucose levels, higher insulin levels and higher insulin secretion indexes upon coffee intake (90). Furthermore, a recent systematic review and meta-analysis showed that green coffee supplementation significantly decreased levels of fasting glucose, insulin, and triglyceride, and improved HDL levels, thus positively affecting the cardiometabolic risk factors (91). Nikpayam et al. (92) also demonstrated a similar effect of green coffee extract on fasting glucose levels and HOMA-IR. Chlorogenic acid, the most abundant biologically active dietary polyphenol in coffee, has been suggested to be responsible for alleviating several cardiometabolic risk factors (93). These beneficial anti-diabetic, anti-obesity, anti-inflammatory and anti-carcinogenic effects seem to be further accompanied by immunomodulatory effects and changes in gut microbiota (94). Interestingly, derivatives of chlorogenic acid, including 3,4-O-dicaffeoyl-1,5-γ-quinide, showed *in vitro* a potent antiviral effect against respiratory syncytial virus, probably acting on an intracellular post-entry replication step (95). Thus, these data suggest that drinking coffee may represent an additional, beneficial strategy in treating viral disease. However, based on our knowledge, it appears that these studies have not been performed *in vivo* yet.

In line with described antidiabetic effects of tea polyphenols, a recent study performed in about 130.000 Korean adults found that increased coffee consumption (more than four cups per day) was associated with a lower prevalence of metabolic syndrome, as compared to non-coffee consumers (96). Another recent study also performed in Korean populations demonstrated that moderate to high coffee intake is inversely associated with the metabolic syndrome (97). Similarly, positive effects of coffee consumption were also demonstrated in Polish population, in which coffee intake was inversely associated with a risk of metabolic syndrome (98–100) and T2D (101). Furthermore, recent studies performed in Japan and Brazil, showed that decaffeinated coffee consumption improved insulin sensitivity in healthy men (102, 103). Another study from Sweden showed an advantage of filtered over boiled coffee when it comes to the protective role of coffee on T2D development (104). It was suggested that long-term consumption of coffee beverages is associated with a lower risk of T2D, probably via multiple mechanisms, including effects on glucose homeostasis, oxidative stress and inflammation (105). Therefore, it would be pertinent to study the potential effects of coffee intake in management and prevention of viral disease, including COVID-19, which seem not be studied *in vivo* yet. At the same time, it would also be important to evaluate further the potential detrimental effects of coffee intake, for example, its effects on sleep quality (106) and endothelial vascular function (107, 108), which may also affect COVID-19 outcome.

## Conclusion

Thus, apart from existing general preventive and safety measures recommended to the general public, maintaining physical activity and choosing a diet and beverages rich in polyphenols, such as tea and coffee, might be important interventions in achieving an optimal public health and offering more efficient and enjoyable way to deal with the COVID-19 pandemic. Particularly, the potential positive effects of coffee consumption as an adjuvant therapy for preventing and managing COVID-19 seem not to be elucidated enough, so further studies are warranted to confirm these beneficial properties.

## Author Contributions

SS conceptualized the topic, researched and analyzed the literature, and wrote the manuscript, including interpretations. FS analyzed background literature and contributed to the conception of the topic, manuscript draft and interpretation, and revised the manuscript critically for its intellectual content. All authors approved the final version of the manuscript.

## Conflict of Interest

The authors declare that the research was conducted in the absence of any commercial or financial relationships that could be construed as a potential conflict of interest.
